# Predictors of the CD24/CD11b Biomarker among Healthy Subjects

**DOI:** 10.3390/jpm11090939

**Published:** 2021-09-21

**Authors:** Shiran Shapira, Gal Aiger, Amitay Ohayon, Dina Kazanov, Fatin Mdah, Marina Ben Shimon, Mori Hay-Levy, Lian Banon, Ido Laskov, Jacob Mashiah, Shahar Lev-Ari, Nadir Arber

**Affiliations:** 1 Integrated Cancer Prevention Center, Tel Aviv Medical Center, Tel Aviv 6423906, Israel; shiransha@tlvmc.gov.il (S.S.); galai@tlvmc.gov.il (G.A.); amitay.study@gmail.com (A.O.); dianak@tlvmc.gov.il (D.K.); mdahfatin@gmail.com (F.M.); marinabs@tlvmc.gov.il (M.B.S.); morih@tlvmc.gov.il (M.H.-L.); shaharl@tlvmc.gov.il (S.L.-A.); 2 Sackler Faculty of Medicine, Tel Aviv University, Tel Aviv 6423906, Israel; jacobm@tlvmc.gov.il; 3 Tel Aviv Medical Center, The Gastroenterology Institute, Tel Aviv 6423906, Israel; lianb@tlvmc.gov.il; 4 Gynecologic Oncology Unit, Tel Aviv Medical Center, Tel Aviv 6423906, Israel; idol@tlvmc.gov.il; 5 The Pediatric Dermatology Unit, Tel Aviv Medical Center, Tel Aviv 6423906, Israel

**Keywords:** CD24, prevention, screening test, biomarker

## Abstract

The CD24 gene has raised considerable interest in tumor biology as a new prognostic factor and a biomarker for the early detection of cancer. There are currently no studies that assess predictors of CD24 in blood tests among healthy individuals. Our aims were (1) to evaluate predictors of the CD24/CD11b biomarker among healthy subjects and (2) to assess CD24/CD11b levels of participants with and without benign tumors. Our cohort included 1640 healthy subjects, aged 20–85, recruited at the Health Promotion and Integrated Cancer Prevention Center (ICPC) in the Tel Aviv Medical Center. Eligible subjects completed a detailed questionnaire on medical history and other epidemiologic information. CD24/CD11b expression in peripheral blood leukocytes (PBLs) obtained from blood samples of participants was analyzed by flow cytometry. Our results showed that the average levels of CD24/CD11b in healthy patients (22.8 ± 9.3) was statistically significant lower compared to subjects with benign cancers (26.1 ± 10.5, *p* < 0.001). Our multivariable analysis demonstrated that elevated levels of CRP (coefficient β: 1.98, *p* = 0.011) were significantly associated with high levels of CD24/CD11b expression among healthy participants. Other risk factors of cancer were not associated with elevated CD24 levels among healthy subjects. In conclusion, our findings may assist in further development and optimization of the CD24/CD11b biomarker to serve as a cancer screening test for early detection of cancer among the healthy population.

## 1. Introduction

Tumor biomarkers are used to describe many different potential markers of cancer development and progression. Ideally, they should be specific to a particular type of cancer and not present in healthy individuals [1]. With the advance of new technologies, many new biomarkers have been developed and are presently undergoing evaluation for the early diagnosis of cancer. In recent years, the CD24 gene has raised considerable interest in tumor biology as a new prognostic factor and as a biomarker for the early detection of cancer [2,3,4,5].

CD24 is a small, mucin-like GPI-anchored receptor. The glycosylphosphatidylinositol-anchored membrane protein CD24 functions as an adhesion molecule between P selectin and L1 and performs a diverse array of functions, including in adaptive immunity, inflammation, autoimmunity, and tumorigenesis [6]. It is involved in cell adhesion and metastasis [6,7]. There is extensive research on the correlation between CD24 and cancer [8,9,10,11,12]. Many studies point to the role of CD24 in tumor growth and cancer progression [6,13,14,15]. CD24 expression has been evaluated as a prognostic marker of poor survival in many types of cancer [14,16,17,18]. CD24 is highly expressed in ovarian, breast, prostate, bladder, kidney, non-small cell, colorectal, and other human cancers [11,15]. Based on these findings, CD24 is being investigated as a marker for early detection of cancer [2,9,19].

We have previously reported that evaluating CD24 levels in peripheral blood leukocytes (PBLs) can serve as a potential promising screening tool for the early detection of colorectal neoplasia and hematological cancers [2,3,4,5]. There is currently a lack of studies that assess predictors of CD24 testing among healthy individuals. Our study aims were (1) to evaluate the association between CD24/CD11b expression and clinical measurements (background diseases, past cancer, family history of cancer, obesity), healthy behaviors (smoking, physical activity), and demographic characteristics (age, gender) among healthy participants and (2) to assess CD24/CD11b levels of participants with and without benign tumors.

## 2. Materials and Methods

### 2.1. Subjects

A total of 1640 blood samples were obtained from healthy volunteers aged 20–85, at the Health Promotion and Integrated Cancer Prevention Center (ICPC) of the Tel Aviv Medical Center. Participants were randomly chosen from the database of the Health Promotion Center. Eligible subjects completed a detailed questionnaire on medical history and other epidemiologic information, including personal and family history of cancer, background diseases, body mass index (BMI), physical activity, smoking status, and demographic characteristics. The complete blood count and inflammatory markers (C-reactive protein and fibrinogen) were evaluated in all participants. Benign cancers identified during the workup were classified according to the site and stage in which they were found. Patients diagnosed with neoplastic lesions were excluded from the study. Blood specimens were drawn using a standard operating procedure, ensuring uniform handling and collection. Patient information was de-identified and only anonymized data was available to the investigators. Written informed consent was obtained from all eligible participants prior to entry into the study. Approval for this study was provided by the Institutional Review Board (IRB, number 05-347) of the Tel Aviv Sourasky Medical Center and the Israeli Ministry of Health.

### 2.2. Isolation of Peripheral Blood Leukocytes

Blood was collected into standard 9 mL collection tubes (Vacuette^®^, Greiner bio-one, cat. No455036). All samples were collected and processed in an identical manner. Peripheral blood leukocytes were isolated from whole blood samples by collecting buffy coats obtained after blood centrifugation for 3 min at 3000 rpm and discarding the plasma supernatant. Residual erythrocytes were lysed by brief incubation in erythrocyte lysis buffer (ELB) containing 155 mM of NH_4_Cl and 0.1 mM of EDTA, followed by 30–40 min on ice in 10 mM of KHCO_3_. Samples were then centrifuged at 3000 rpm at 4°C for 5 min. Clean leukocyte pellets were obtained after 1–2 more washes with ELB. PBLs were than fixated with formaldehyde (FA) solution (2% FA in PBS) for 15 min at room temperature (RT) and stored at 4°C until use. The fixed cells were stored for up to 5 days. We performed a validation test under GLP conditions, which included a comparison between fresh samples and those stored after fixation. This validation study confirmed the stability and repeatability of the test results under these conditions. All samples underwent the same processing.

### 2.3. Flow Cytometry

A total of 1 × 10^6^ leukocytes were used for each test and stained with 0.05 µg anti-CD24-FITC mAb (NS17), anti-CD11b-PerCp-Cy5.5, or remained unstained for 30 min at RT. The cells were then washed twice with FACS buffer (0.01% sodium azide, 10% fetal bovine serum [FBS] in ice-cold PBS) and analyzed by flow cytometry (CyFlow Cube 6, Sysmex, Germany). Data were analyzed following the creation of a hierarchical population tree in the software at the beginning of the screen. This template was used in all subsequent analyses. The template file included compensation adjustment, which was uniformly applied to all the collected data to minimize fluorescence overlap between detection channels. The percentage of positive cells was determined by subtracting the percentage of CD24 and CD11b-positive cells (dual stain) from CD24-positive cells (single stain). The percentage of positive cells was determined by subtracting the percentage of CD24 and CD11b-positive cells (dual stain) from CD24-positive cells (single stain), as has been published previously [5].

### 2.4. Statistics

Descriptive statistics (N, mean, standard deviation, proportion) were calculated for study variables; these are presented in tables as well as graphically via box plots by benign cancer/healthy status. An independent-samples *t*-test was used to compare between CD24/CD11b expression of subjects with or without benign cancer. Linear regression was performed to assess association between CD24/CD11b expression and predicting variables. To take into account the presence of multicollinearity, a variance inflation factor (VIF) was calculated for all independent variables; a VIF larger than 5 suggests the detection of multicollinearity [20]. All tests were two-tailed and *p* < 0.05 were considered significant. The statistical software R (4.0.2) and SPSS 27 (IBM, New York, NY, USA) were used for all the data processing within this study.

## 3. Results

### 3.1. Demographics and Population Characteristics

Population characteristics of a cohort of 1640 healthy subjects are presented in Table 1. Six hundred sixty-eight (44.8%) of the subjects were female. The mean age was 48.7 (SD = 12.3). A total of 742 (54.2%) had a first- or second-degree relative with a history of cancer before the age of 70, and 310 (24.8%) of the patients smoked. The average level of CRP was 6.3 (SD = 20.8); 134 of the subjects (10.0%) were shown to have elevated CRP levels (above 10 mg/mL).

### 3.2. CD24 Levels in Subjects with Benign Tumors Compared to Healthy Subjects

**Figure 1:**
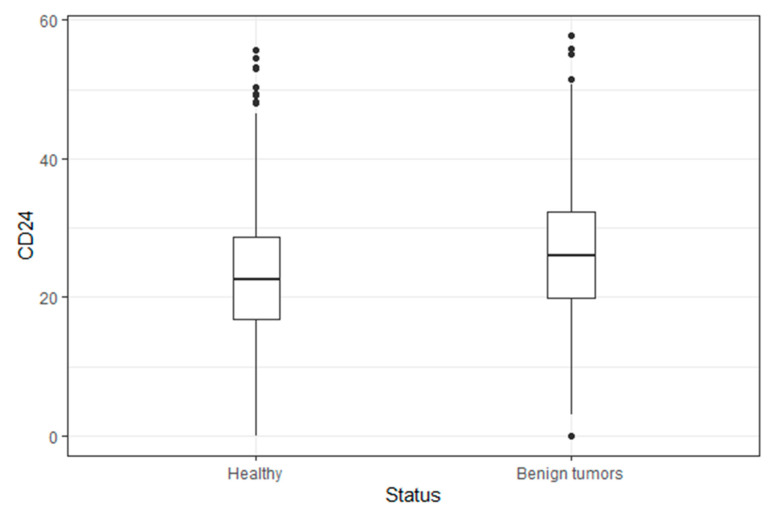
Box plot of the CD24 levels in healthy subjects versus subjects with benign tumors. Flow cytometry analysis showed levels of CD24/CD11b expression in PBLs obtained from study participants.

CD24/CD11b expression was analyzed in 1640 participants (23.4 ± 9.7), of which 297 had benign tumors. Figure 1 presents a box plot of the CD24/CD11b levels in healthy subjects versus subjects with benign cancer. Flow cytometry analysis showed levels of CD24/CD11b expression in PBLs obtained from the study participants. The average level of CD24/CD11b in healthy patients (22.8 ± 9.3) was significantly lower compared to the that of the subjects with benign tumors (26.1 ± 10.5, *p*<0.001). Benign tumor type distribution is attached as Supplementary Table S1.

### 3.3. Association between Participants’ Characteristics and CD24 Levels Univariate and Multivariate Analysis

We evaluated the association between CD24/CD11b expression and the subject’s clinical measurements (background diseases, past cancer, family history of cancer, being overweight), healthy behaviors (smoking, physical activity), and demographic characteristics (age, gender). Table 2 shows CD24/CD11b levels of study participants across the different measurements.

Our multivariate analysis demonstrated that elevated levels of CRP (coefficient β: 1.98, *p* = 0.011) was significantly associated with high levels of CD24/CD11b expression (Table 3). Age, being overweight, background disease, physical activity, and past cancer were positively associated with CD24/CD11b expression in the univariable analysis, but no association was found after multivariable adjustment. Other variables were not associated with CD24/CD11b expression in both our univariable and multivariable analyses.

Age was correlated with past cancer (*p* < 0.001), background diseases (*p* < 0.001), and physical activity (*p* = 0.001). These variables were not found to have high VIF in our multicollinearity analysis (past cancer, VIF = 1.13; background diseases, VIF = 1.20; physical activity, VIF = 1.05); therefore, they were included in the multivariable analysis (the multicollinearity analysis is attached as Supplementary Table S2).

## 4. Discussion

This is the first study to assess predictors for CD24 expression among healthy subjects. Our study demonstrated that elevated CRP levels are associated with higher CD24/CD11b levels in the PBLs of healthy participants. In addition, there was a significant difference between the expression of CD24/CD11b levels in participants with benign tumors as compared to healthy subjects. Other risk factors of cancer were not found to be correlated with CD24/CD11b levels among healthy participants.

Several studies have shown that increased expression of CD24 is associated with the early stages of tumorigenesis [2,3,13,21,22]. In our study, we found elevated levels of CD24 in participants with benign tumors compared to the CD24 levels of healthy subjects (Figure 1). Our results are consistent with other studies demonstrating that CD24^+^ positive cells have a high capability to form tumors [23,24,25]. CD24 was shown to play an important role in cells’ proliferation, adhesion, and regulation of angiogenesis [23,24,25,26,27,28,29]. These processes are key cellular events involved in the formation of benign or cancerous tumors [30,31,32]. Our previous studies have shown that CD24 levels were elevated in transformed enterocytes compared to normal cells, and that CD24 is overexpressed in colonic mucosa already at an early stage of colorectal cancer carcinogenesis [2,3]. These findings indicate the rationale for assessing CD24 as a possible biomarker for the detection of early-stage malignancies.

In our study, healthy subjects with high levels of CRP were found to have higher CD24 levels (Table 3). The association remained robust and significant both in our univariate and multivariate analyses. Our findings are consistent with previous studies showing that elevated CD24 levels are associated with inflammation and autoimmune diseases such as inflammatory bowel disease, rheumatoid arthritis, and multiple sclerosis [33,34,35,36]. These chronic inflammatory diseases are also characterized by, among other clinical measures, high CRP levels [37,38,39,40,41,42]. Multiple lines of evidence have found correlations between autoimmune and inflammatory diseases and elevated risk of cancer: IBD was associated with an increased risk of developing colon cancer [43,44], and rheumatoid arthritis was associated with an elevated risk of developing lymphoma [45,46]. Circulating CRP levels may express inflammation in the micro-environment of tumors. This inflammation occurs at the initial steps of tumorigenesis and produces an attractive pro-neoplastic environment for its growth by damaging DNA, promoting angiogenesis and metastatic proliferation [47,48]. Other studies have evaluated the effects of CRP on human lymphocyte responsiveness [49]. Additional studies are warranted to investigate the cellular pathways that connect CRP levels in the blood and higher CD24/CD11b levels in PBLs.

Age is one of the most studied risk factors for cancer [50]. The incidence of most cancers increases with age, rising more rapidly from midlife onwards. The relationship between age and cancer risk is influenced by genetically inherent processes but can also be modified by environmental influences [51,52,53]. The role of epigenetic DNA alterations in the complex aging process is an emerging area of research. Epigenetic changes are known to influence gene expression and are implicated in tumor progression. In our study, age was positively correlated with other risk factors of cancer: background diseases, past cancer, and the lack of physical activity. These variables were associated with CD24/CD11b expression in the univariable analysis, but no association was found after multivariable adjustment (Table 3). Other variables tested were not associated with CD24/CD11b expression in either our univariable or multivariable analysis. These results demonstrate that the variability of CD24 is not dependent on a subject’s clinical measurements (background diseases, past cancer, family history of cancer, being overweight), healthy behaviors (smoking, physical activity), and demographic characteristics. These findings may suggest the stability of CD24 to variations in population characteristics and indicate the feasibility of CD24 as a specific biomarker for cancer.

Our study has several limitations. First, the subjects voluntarily applied to the Center for Preventive Medicine, which is located in the center of the country. While this reflects the sample composition of study subjects at our institution, the generalizability of these results may be limited. Secondly, our information on physical activity and smoking status was limited, and a more detailed categorization of these variables would enable us to analyze the impact of these parameters.

In conclusion, a significant increase in the levels of CD24/CD11b was found among subjects with benign tumors as compared to healthy subjects. Elevated CRP levels were associated with higher CD24/CD11b levels in the PBLs of healthy participants, suggesting that predictors of CD24/CD11b levels among healthy subjects may assist in further development and optimization of the CD24/CD11b biomarker to serve as a cancer screening test for the early detection of cancer among the healthy population.

## Figures and Tables

**Table 1:** Demographic and population characteristics of the participants (N = 1640).

	Characteristic	Study Population N (%) or Mean (SD)
Age At Visit		48.7 (12.3)
Gender (%)	Female	668 (44.8%)
	Male	822 (55.2%)
Smoking (%)	No	939 (75.2%)
	Yes	310 (24.8%)
Overweight (%)	No	993 (83.0%)
	Yes	203 (17.0%)
Background Disease (%)	With	1147 (77.0%)
	Without	343 (23.0)
Family History of Cancer	Without	626 (45.8)
	With	742 (54.2)
Physical Activity (%)	No	333 (28.0%)
	Yes	858 (72.0%)
CRP Levels (mg/mL)	<10	1209 (90.0)
	≥10	134 (10.0)
Past Cancer (%)	No	1404 (94.2%)
	Yes	86 (5.8%)

SD—standard deviation; CRP—C-reactive protein; smoking: have you ever smoked/do you smoke? (yes/no); physical activity: do you exercise at least once a week? (yes/no). Clinical variables were collected from medical files—family history of cancer: first- or second-degree relatives under 70 that had cancer; past cancer; background disease: indexed according to the Charlson Comorbidity Index (CCI, ICD-9-CM index); overweight: BMI above 25.

**Table 2:** CD24/CD11b levels of study participants across the different measurements.

	Characteristic	CD24/CD11b Levels Mean (SD)
Age	(19–85)	22.9 (9.4)
Gender (%)	Female	22.4 (9.6)
	Male	23.2 (9.3)
Smoking (%)	No	22.4 (9.3)
	Yes	22.7 (9.7)
Overweight (%)	No	23.8 (9.0)
	Yes	22.2 (9.3)
Background Disease (%)	With	24.7 (9.4)
	Without	22.5 (9.4)
Family History of Cancer	Without	22.4 (9.5)
	With	22.7 (9.1)
Physical Activity (%)	No	23.4 (10.0)
	Yes	22.0 (9.1)
CRP Levels (mg/mL)	<10	22.3 (9.3)
	≥10	27.2 (9.8)
Past Cancer (%)	No	22.6 (9.4)
	Yes	26.6 (10.0)

SD—standard deviation; CRP—C-reactive protein; smoking: have you ever smoked/do you smoke? (yes/no); physical activity: do you exercise at least once a week? (yes/no). Clinical variables were collected from medical files—family history of cancer: first- or second-degree relatives under 70 that had cancer; past cancer; background disease: indexed according to the Charlson Comorbidity Index (CCI, ICD-9-CM index); overweight: BMI above 25.

**Table 3:** Association between participants’ characteristics and CD24 levels for univariate and multivariate analyses.

	CD24/CD11b Levels			
Participants’ Characteristics	Univariable Analysis		Multivariable Analysis	
	β	*p*	β	*p*
Age	0.07	0.002	0.04	0.130
Gender (Female vs. Male)	0.75	0.147	0.91	0.104
Smoking (Smoking vs. Non-Smoking)	0.29	0.651	0.19	0.783
Overweight (Yes vs. No)	1.63	0.027	0.85	0.295
Background Disease	2.25	<0.001	0.30	0.716
Family History of Cancer (Yes vs. No)	0.34	0.517	0.56	0.313
Physical Activity (%)	−1.36	0.028	−1.09	0.097
CRP (Normal vs. Elevated)	4.91	<0.001	3.97	0.001
Past Cancer (%)	4.00	<0.001	0.79	0.655

β, standardized coefficient; SD—standard deviation; CRP—C-reactive protein; smoking: have you ever smoked/do you smoke? (yes/no); physical activity: do you exercise at least once a week? (yes/no). Clinical variables were collected from medical files—family history of cancer: first- or second-degree relatives under 70 that had cancer; past cancer; background disease: indexed according to the Charlson Comorbidity Index (CCI, ICD-9-CM index); overweight: BMI above 25.

## Data Availability

The data presented in this study are available on request from the corresponding author.

## Supplementary Materials

The following are available online at https://www.mdpi.com/article/10.3390/jpm11090939/s1.
