# Supplementary material for: Predictors of the CD24/CD11b Biomarker among Healthy Subjects
Source: J Pers Med. 2021 Sep 21;11(9):939. doi: 10.3390/jpm11090939 (PMC8471999; doi:10.3390/jpm11090939)
Supplement: Supplementary file 1 [file jpm-11-00939-s001.zip › jpm-1364710-supplementary.pdf]

Supplementary Table S1. Benign tumor distribution

	Percent
Benign tumor of colon and rectum	18.5
Benign neoplasm of skin	10.7
Benign neoplasm of thyroid	8.2
Benign tumor of esophagus	1.1
Benign tumor of liver	5.0
Benign tumor of prostate	5.0
Benign tumor of bladder	3.9
Benign tumor of breast	7.5
Benign tumor of kidney	3.6

Supplementary Table S2. The result for multicollinearity among variables in the multivariate logistic regression model

	Tolerance	Variance Inflation Factor (VIF)
Age	0.883	1.133
Gender (Female vs Male)	0.972	1.029
Smoking (Smoking vs Non Smoking)	0.963	1.039
Overweight (Yes vs No)	0.911	1.098
Background Disease	0.843	1.187
Family History of Cancer (Yes vs No)	0.984	1.017
Physical Activity (%)	0.955	1.048
CRP (Normal vs Elevated)	0.942	1.061
Cancer in the past (%)	0.890	1.124
